# Differences in* Rhodococcus equi *Infections Based on Immune Status and Antibiotic Susceptibility of Clinical Isolates in a Case Series of 12 Patients and Cases in the Literature

**DOI:** 10.1155/2016/2737295

**Published:** 2016-08-11

**Authors:** Praveen Gundelly, Yasuhiro Suzuki, Julie A. Ribes, Alice Thornton

**Affiliations:** University of Kentucky, Lexington, KY 40536, USA

## Abstract

*Rhodococcus equi* is an unusual zoonotic pathogen that can cause life-threatening diseases in susceptible hosts. Twelve patients with* R. equi *infection in Kentucky were compared to 137 cases reported in the literature. Although lungs were the primary sites of infection in immunocompromised patients, extrapulmonary involvement only was more common in immunocompetent patients (*P* < 0.0001). Mortality in* R. equi*-infected HIV patients was lower in the HAART era (8%) than in pre-HAART era (56%) (*P* < 0.0001), suggesting that HAART improves prognosis in these patients. Most (85–100%) of clinical isolates were susceptible to vancomycin, clarithromycin, rifampin, aminoglycosides, ciprofloxacin, and imipenem. Interestingly, there was a marked difference in susceptibility of the isolates to cotrimoxazole between Europe (35/76) and the US (15/15) (*P* < 0.0001). Empiric treatment of* R. equi* infection should include a combination of two antibiotics, preferably selected from vancomycin, imipenem, clarithromycin/azithromycin, ciprofloxacin, rifampin, or cotrimoxazole. Local antibiograms should be checked prior to using cotrimoxazole due to developing resistance.

## 1. Introduction 

*Rhodococci* are aerobic, Gram positive, pleomorphic, and nonmotile bacteria, which can be detected in soil and grow well on simple nutrients provided by herbivore manure. They also grow well in the intestines of grazing animals. Among the organisms making up the genus* Rhodococcus*,* R. equi* is the most common isolate and has been well recognized as an important pathogen in veterinary medicine. It can cause bronchopneumonia, especially in foals [1].* R. equi* was first isolated from foals with bronchopneumonia in 1923 [2]. The genus* Rhodococcus* is now known to be distinct from the other closely related species of acid-fast or modified acid-fast organisms of the genera* Gordonia*,* Nocardia,* and* Mycobacterium* [3].

The vast majority of human infections with the* Rhodococcus* spp. are caused by* R. equi* [4], whereas there have been case reports of human infection by the other species [5–7]. The first human case of* R. equi* infection was described by Golub et al. in 1967 in a 29-year-old male with plasma cell hepatitis on chronic steroid therapy who presented with cavitary pneumonia [8]. Frequency of infection with* R. equi* increases in immunocompromised individuals such as those with AIDS and organ transplants [9]. Since the first case of* R. equi* infection in an AIDS patient in 1986 [10], there have been increasing numbers of infections with this bacterium reported in AIDS patients [9]. Emergence of resistance to macrolides and rifampin has been well documented in* R. equi* isolated from animals. However, there is no systematic information available regarding antibiotic resistance of* R. equi *isolates from humans. In addition, there is no standard antibiotic susceptibility testing panel for* R. equi* in humans. The main objective of the present study is to summarize disease characteristics and antibiotic susceptibility testing results in 12 cases of* R. equi* infection at a tertiary care center in Central Kentucky and compare with the cases of the infection with this pathogen in the United States and Europe reported in the literature to generate systematic information on clinical diseases associated with* R. equi* infection and antibiotic susceptibility of the bacterium isolated from the patients.

## 2. Methods 

### 2.1. Identification of Patients with* R. equi* Infection at the University of Kentucky

Following IRB approval, the microbiology laboratory database was used to identify subjects from whom* R. equi* was isolated in the period from January 1998 to December 2013. A SUNQUEST Epi report was generated searching for* Rhodococcus equi *in the result field. Other information collected in this report included subject name, medical record number, specimen source, date of specimen collection, and antibiotic susceptibility test results for the isolate. A total of 14 subjects were identified from whom* R. equi* was isolated. Among these isolates, the results of antibiotic susceptibility testing were available for 11 subjects. Chart review was performed on 12 of the 14 subjects to gather age, gender, occupational exposure to animals, HIV status, viral load, CD4 count, clinical and radiographic features, and antibiotic treatment and outcomes. Two subjects were from a referral hospital and their medical records were not available for review.

Identification of* R. equi* was performed by BD Phoenix*™* Automated Microbiology System (Becton, Dickinson and Company, New Jersey). Screening of antibiotic susceptibility for these clinical isolates was performed by Epsilometer-test (E-test) (BioMérieux, Durham, NC). The interpretive criterion for determining susceptibility to antibiotics followed the CLSI Guidelines in the M100 document for* Staphylococcus aureus*, with the inclusion of results for vancomycin and rifampin [17]. Break points for ciprofloxacin and levofloxacin were interchangeable. Clarithromycin was used as a class representative for newer macrolides.

### 2.2. Literature Search on Cases of* R. equi* Infection

PubMed search was performed using key words “*Rhodococcus equi”* and “*Corynebacterium equi”* (an older, now obsolete name for the organism). The articles containing these key words were then filtered by using additional key words “Humans” and “English language”. Abstracts of these studies were reviewed and any study describing at least 10 cases was included in this study. There were four European studies [11–13, 16] and two United States (US) studies [14, 15] that satisfied this requirement. Antibiotic susceptibility data were not available in one of the two US studies [15].

### 2.3. Statistical Analyses

Statistical significances of the differences in clinical features between groups of patients and in antibiotic susceptibility of clinical isolates were determined by Fisher's exact test. In the analyses of the differences in antibiotic susceptibility, “corrected” *P* values were calculated by multiplying each *P* value by the number of antibiotics studied [18].

## 3. Results

### 3.1. Case Series at the University of Kentucky

There were twelve cases in this series including six patients positive for human immune deficiency virus (HIV), two with organ transplant, and four immune competent individuals (Table 1). Two of the immune competent patients had wound infection with* R. equi* and the other two had pneumonia. All eight immunocompromised patients had pneumonia.* R. equi*-infected patients presented with varying symptoms depending on organ system involved (Table 1). Most common symptoms were fever, cough, dyspnea, anorexia, and weight loss in patients diagnosed with pneumonia.* R. equi* was isolated from respiratory specimens in 7 patients and from the peripheral blood from 5 patients.* R. equi* was also isolated from wound cultures from two immunocompetent individuals.

Among the 10 cases with* R. equi* pneumonia, bacteremia was noted in five patients, one immunocompetent and four immunocompromised. A cavitary lesion was noted in radiographic imaging in 4 of the 10 patients. Three of these patients had upper lobe cavitary lesions and one had lower lobe cavitation. The presence of the cavitary lesion did not correlate with the presence of bacteremia. All six patients with HIV infection were not on antiretroviral therapy at the time of diagnosis of* R. equi* infection; 5 of them were antiretroviral therapy (ART) naïve and the other patient stopped ART a few months prior to diagnosis of* R. equi* infection. ART was initiated in all the patients after the diagnosis of* R. equi* infection at outpatient follow-up. One patient was consistently nonadherent with ART and other medications and finally succumbed to infection after one year of initial diagnosis of* R. equi* pneumonia.

In the two organ transplant patients with* R. equi* infection, one had received a lung transplant and the other had received a renal transplant. Both the transplant patients were treated successfully with prolonged course of antibiotics and they did not have any recurrence of infection.

In the immunocompetent patients, treatment of the two individuals with wound infection was successful. The other two individuals with* R. equi* pneumonia did not follow up in the university health system. Based on social security death index data, one patient died after 6 months and the other patient expired after 15 months, both from unknown causes.

### 3.2. Review of Clinical Characteristics of Infection with* R. equi* in This Study and Cases in the Literature

The epidemiological and clinical characteristics of subjects infected with* R. equi* were analyzed for four European studies [11–13, 16] and three US studies including two studies in the literature [14, 15] and the present study.

In the four European studies, all 113 patients were immunocompromised with HIV infection. Among these patients, 107 subjects (95%) had pulmonary disease with or without extrapulmonary* R. equi* infection, and six subjects had extrapulmonary involvement only (Table 2). In the three US studies including the present study, 21 immunocompromised subjects (95%) had pulmonary diseases with or without extrapulmonary involvement and one subject had extrapulmonary involvement only (Table 2). Therefore, there is no significant difference in the organ system involvement in infection with* R. equi* in the immunocompromised patients between the European and US subjects.

In contrast, in the cases of* R. equi* infection in immunocompetent individuals in the US, only 5 of 14 patients had pulmonary diseases with or without extrapulmonary involvement, and the other 9 patients had extrapulmonary involvement only (Table 2). Extrapulmonary sites of infection included central nervous system, bone, blood, eyes, and soft tissue. The frequency of extrapulmonary involvement only in the immunocompetent patients (9/14 cases) was significantly higher when compared to the frequencies of those in the immunocompromised patients in the US (1/21) and those (6/113) in European studies (*P* = 0.0002 for US and *P* < 0.0001 for European studies; Table 2).

In patients with pneumonia, among the 107 subjects in the European studies, 74 (69%) had cavitary lesions in the lungs (Table 3). Similarly, 17 of 26 subjects (65%) in the US studies who were diagnosed with pneumonia presented with cavitary lesions (Table 3).

The outcome of* R. equi* infection in HIV-positive patients depended strongly upon treatment of HIV with highly active antiretroviral therapy (HAART). Among the total 113 HIV-positive subjects in the European studies, 93 were diagnosed in the pre-HAART era and 20 were diagnosed in the HAART era. Mortality was 56% (52/93) in pre-HAART era while it is only 5% (1/20) in HAART era (Table 4, *P* < 0.0001). The outcome in two additional subjects diagnosed with* R. equi* infection in pre-HAART era was unknown. Therefore, HAART significantly improved survival in HIV-positive patients with* R. equi* infections. A similar tendency was observed in the US studies. Among 12 HIV-positive patients diagnosed with* R. equi* infection in pre-HAART era, 7 of them (58%) died, whereas mortality was only 1 of 6 patients (17%) diagnosed in HAART era (Table 4). However, the difference in the mortality between pre-HAART and HAART era in the US studies did not reach statistical significance most likely due to the small number of patients available in the analysis. When the data from the European and US studies are combined, mortality in* R. equi*-infected HIV patients was higher in the pre-HAART era (56%, 59/105) than in HAART era (8%, 2/26) (*P* < 0.0001).

### 3.3. Review of Antibiotic Susceptibility of* R. equi* Isolated from Patients in the Present Study and in the Literature

The method of antibiotic susceptibility testing and minimum inhibitory concentration (MIC) breakpoints assessment are not described in all of the 6 studies that were analyzed. E-test was used in the study by Arlotti et al. [11] and the present study at the University of Kentucky. There were significant differences in the panel of antibiotics chosen for susceptibility testing as these studies span over two decades. Antibiotics for which susceptibility testing was performed in most studies included vancomycin, erythromycin/clarithromycin, cotrimoxazole, rifampin, penicillin, ciprofloxacin, and gentamicin. Twenty-one antibiotics were selected for which susceptibility data were available for at least 10 isolates of* R. equi*. These are listed in the order of higher susceptibility in Table 5. At least 85% of* R. equi* isolates were susceptible to 10 of the 21 antibiotics evaluated. These ten antibiotics are clarithromycin, vancomycin, rifampin, amikacin, erythromycin, teicoplanin, imipenem, netilmicin, gentamicin, and ciprofloxacin. In contrast,* R. equi* isolates were consistently resistant to penicillin, oxacillin, ampicillin, cephalothin, and clindamycin, and their susceptibilities to these antibiotics were lower than 15% (Table 5).

In most of the 21 antibiotics listed in Table 5, susceptibility of the bacterium was similar among 6 studies including the present study. This strongly suggests that methodological differences in testing antibiotic susceptibility of* R. equi* isolates in these 6 studies did not significantly affect the outcome of the tests. However, there is a marked difference in susceptibility of the clinical isolates to cotrimoxazole between European and US studies. One hundred percent (15/15 isolates) of* R. equi* from the US were susceptible to cotrimoxazole, whereas only 35 of 76 isolates (46%) from Europe were susceptible to this compound (*P* < 0.0001, corrected *P* < 0.0018). A similar tendency was also observed in susceptibility of the bacterium to tetracycline. Nine of the 10 isolates (90%) from the US were susceptible to tetracycline, whereas only 17 of 42 (43%) of isolates from Europe were susceptible to this antibiotic (*P* = 0.0127). However, this difference did not reach statistical significance when corrected for the number of variables tested (corrected *P* = 0.229). The difference in susceptibility of the* R. equi* isolates to cotrimoxazole was not due to differences in the sites of isolation of the bacterium from the patients in the European and US studies, because a majority of the isolates were from either the respiratory specimen (48% in Europe and 45% in the US) or the peripheral blood (34% in Europe and 36% in the US) (Table 3).

### 3.4. Empiric Treatment of* R. equi* Infection

Based on antibiotic susceptibility of the clinical isolates shown in Table 5,* R. equi* infection in immunocompromised patients and the serious infection in immunocompetent individuals should receive intravenous therapy with 2 or 3 drugs that include vancomycin, imipenem, clarithromycin/azithromycin, rifampin, aminoglycoside, or ciprofloxacin/levofloxacin [9, 19]. Most patients require a minimum of 2 weeks of intravenous therapy at which point oral therapy with 2 or 3 drugs that include vancomycin, rifampin, or ciprofloxacin/levofloxacin may be substituted based on clinical improvement [9, 20]. Immunocompetent patients with mild to moderate diseases can be treated with two active oral agents [19]. Vancomycin and imipenem are bactericidal agents that help decrease high bacterial burden and rapid clearance of bacteremia [9, 16]. Clarithromycin, rifampin, ciprofloxacin, and vancomycin achieve good intracellular concentrations and can be very effective on intracellular pathogens [15, 19]. Cotrimoxazole is an option for both intravenous and oral therapy but is recommended for empiric therapy in the US but not in Europe due to the high frequency of resistance of the* R. equi* isolates to this antibiotic in Europe as described earlier.

## 4. Discussion

*R. equi* has been known to cause pneumonia in immunocompromised individuals since it was first reported in 1967 [8]. In the literature, 95–100% of immunocompromised patients infected with the bacterium presented with pulmonary involvement [11, 16]. In agreement with these findings, the cases identified in Kentucky also presented with pulmonary involvement for all eight immunocompromised subjects. This was not the case for immunocompetent patients, however. Only two of four (50%) immunocompetent subjects in the currently described patient series had pulmonary involvement. A similar tendency can be seen in other studies in the US, in which pneumonia was observed only in 3 of 10 (30%) infected immunocompetent patients [14, 15]. Therefore, it is important to recognize that immunocompetent subjects may not present with pneumonia when infected with* R. equi* but instead may have localized infections such as cutaneous wounds or osteomyelitis.

Since pulmonary involvement was observed in the majority of immunocompromised but not of immunocompetent patients infected with* R. equi*, insufficient immune responses appear to be a significant cause that makes individuals susceptible to pulmonary infection with this bacterium. In immunocompetent individuals, a local exposure of organ(s) such as skin or a bone to the materials, possibly soil, highly contaminated with* R. equi* may have caused these individuals to be susceptible to the infection.

In a review of the literature, cavitary lesions were detected on radiologic imaging in 40–88% (mean 68%) of subjects diagnosed with* R. equi* pneumonia. In the case series at the University of Kentucky, 3 of 4 (75%) of patients with cavitary lesions had the lesions of an upper lobe. Similar tendencies were observed in other case series describing radiologic findings in* R. equi* pneumonia [21, 22]. Therefore, the presence of upper lobe cavitary lesions appears to be an important differential feature of* R. equi* infection by imaging.

In this review, mortality of HIV patients infected with* R. equi* is 56% in the pre-HAART era while it was only 8% in HAART era. The study by Torres-Tortosa et al. [16] showed improved survival in patients in whom HAART was started. These results strongly suggest that the protective immune responses are crucial, in addition to antibiotic treatment, to eradicate* R. equi* and prevent mortality.

Studies using animals infected with* R. equi *have demonstrated the importance of both cell-mediated and humoral immune responses in clearing the bacterium [23]. It is well recognized that innate immune cells such as neutrophils and macrophages are crucial to control primary infection [24]. Th1-type immune responses (IL-2, IL-12, IFN-*γ*, and TNF-*α*) are important for resistance to* R. equi*. Both CD4 and CD8 T cells produce IFN-*γ* which is necessary to activate macrophages to eliminate* Rhodococcus* from intracellular locations.* Rhodococcus-*specific antibodies opsonize the extracellular organisms to promote phagocytosis and enhance bacterial killing [25]. This antibody-mediated protective mechanism is relevant because* R. equi* is a facultative intracellular bacterium. For example, newborn foals are exposed to* R. equi* infection soon after birth as it is ubiquitous in soil. However, most of them do not develop clinical infection until 6–12 weeks after birth, which coincides with decreases of maternal antibodies in these animals.

In agreement with the importance of the protective immunity to control* R. equi* in animal models, immunocompetent patients with* R. equi* infection treated with antibiotics have better outcomes in general when compared to infected immunocompromised individuals [15, 26–29]. However, there are case reports of deaths in immunocompetent patients diagnosed with* R. equi* pneumonia [30, 31]. In the current case series, two immunocompetent patients with* R. equi* pneumonia died after six and fifteen months of discharge from hospital, respectively, although the cause of death in these patients is unknown as they were lost to follow-up. Antibiotic treatment is essential to eliminate* R. equi* infection not only in immunocompromised but also in immunocompetent individuals [30, 31]. Virulent strains of* R. equi* have been shown to contain a plasmid, which encodes seven related virulence associated proteins (Vaps) including the immunodominant surface-expressed protein Vap A that is essential for intracellular growth of the bacterium in macrophages [32, 33]. These virulence proteins may contribute to reducing the efficiency of the protective immunity to control the bacterium.

The present study made an interesting observation with regard to antibiotic susceptibility to cotrimoxazole between the US and Europe. All clinical isolates in the US were susceptible to cotrimoxazole, whereas more than half of the isolates in Europe were resistant. This is the first time that this difference has been reported. This difference in susceptibility to cotrimoxazole may suggest that the US and Europe have different strains of* R. equi*. It is unclear why and how the bacterium in Europe acquired resistance to cotrimoxazole. It is possible that differences in usage of antibiotics in domestic animals between these two continents have resulted in the disparity in susceptibility to the antibiotic.

Based on the consensus findings for antibiotic susceptibility for clinical isolates, an empiric antibiotic treatment regimen for* R. equi* infection would include 2-3 intravenous agents (vancomycin, imipenem, clarithromycin/azithromycin, rifampin, an aminoglycoside, ciprofloxacin/levofloxacin, or cotrimoxazole), administered for at least 2 weeks if the patient is immunocompromised [9, 19], or 2 oral agents (clarithromycin/azithromycin, rifampin, ciprofloxacin/levofloxacin, or cotrimoxazole) if the patient is immunocompetent with mild or moderate diseases [19]. Final antibiotic choice should be determined based on* in vitro* antibiotic susceptibility testing results. Monotherapy for* R. equi *infection is not recommended, as it is often ineffective. This infection should be treated with at least two agents with activity against* R. equi.* Parenteral therapy is generally recommended for administration during the first few weeks of a 6-month or longer course of treatment. One of these agents should be bactericidal given the frequency with which it is isolated from blood [34, 35] and one agent should be active against intracellular organisms.* R. equi* is usually susceptible to erythromycin and extended spectrum macrolides, rifampin, fluoroquinolones, aminoglycosides, glycopeptides, and imipenem [36]. Macrolides, fluoroquinolones, glycopeptides, and rifampin have the additional advantage of achieving good intracellular drug concentration [37].* In vitro* studies showed combinations of gentamicin with erythromycin or rifampicin had antagonistic effects on killing compared to either drug alone, while combinations of erythromycin with rifampicin or penicillin had synergistic effect [38].

In this case series at the University of Kentucky, one in eight clinical isolates was resistant to rifampin. Recent studies have shown emergence of macrolide and rifampin resistant* R. equi* strains in horse breeding farms because of widespread use of these antibiotics for prophylaxis and treatment [39]. Rifampin resistant* R. equi* strains were isolated from HIV patients from northern Thailand [40]. Rifampin resistance is associated with mutations in rpo B gene. In view of this, a combination of vancomycin and macrolide as initial empiric therapy for* R. equi* infections is suggested. Aminoglycosides have good activity against* R. equi*. However, they are falling out of favor because of their toxicity profile. Among carbapenems, more data is available for imipenem than for meropenem [11, 13, 16].

The duration of therapy depends on the site(s) and extent of infection, underlying immunocompetence of the host, and the clinical response to therapy. A minimum of 6 months of antibiotic therapy is typically required for immunocompromised patients with pulmonary, bone and joint, or central nervous system infections [9]. Secondary prophylaxis with an oral agent may be considered in patients who are on immune suppressive agents or HIV patients with low CD4 count [9]. Any improvement in the immunocompetence of the host should be pursued as a therapeutic adjunct in treating* R*.* equi* infections, either through curtailment of immunosuppressive medications or through aggressive antiretroviral therapy.

In the present study, twelve patients with* R. equi *infection in Kentucky were compared to 137 cases reported in the literature. In both the cases in Kentucky and those in the literature, lungs were the primary sites of infection in immunocompromised patients, whereas extrapulmonary involvement only was more common in immunocompetent patients. In* R. equi*-infected immunocompromised patients with HIV, HAART improves prognosis in these patients. Based on antibiotic susceptibility of the bacterium isolated from the patients, the empiric treatment of* R. equi* infection should include a combination of two antibiotics, preferably selected from vancomycin, imipenem, clarithromycin/azithromycin, ciprofloxacin, rifampin, or cotrimoxazole. Local antibiograms should be checked prior to using cotrimoxazole due to developing resistance.

## Figures and Tables

**Table 1:** Demographic and clinical characteristics of *R. equi* infection at the University of Kentucky^*∗*^.

Year	Patient data			Clinical data								
	Case/age/gender	Occupational/environmental exposure to livestock	Specimen source	Clinical symptoms	Immune status	CD4 (%)	Viral load copies/mL	ART	Radiography	Treatment duration (months)	Surgery	Relapse/outcome
2001	1/40/m	Unknown	Sputum, blood	Fever, cough, nausea	HIV	27 (6)	56784	No	RUL infiltrate with cavitation and LUL infiltrate	3	No	Cured

2001	2/32/m	Unknown	Sputum, blood	Fever, nausea, weight loss	HIV	43 (4)	<400	Yes	LUL and LLL pneumonia	12	No	Relapsed^†^/expired

2003	3/33/m	Lawn care worker	Sputum	Fever, cough	HIV	60 (8)	257927	No	LUL cavitary lesion	9	No	Cured

2009	4/40/m	Unknown	Sputum	Cough, dyspnea	HIV	7 (3)	354000	No	Right lung infiltrate	36	No	Cured

2010	5/37/m	Horses	Sputum	Cough, dyspnea	HIV	59 (22)	276000	No	LLL cavitary lesion	24	No	Cured

2013	6/48/m	Horses, donkey	Blood pericardial fluid	Fever, cough, dyspnea	HIV	10 (5)	143000	No	Multiple bilateral nodular lesions	12	Pericardial drain	Cured

2008	7/62/m	Unknown	Sputum	Anorexia, dyspnea, nausea	Lung tx	NA	NA	No	Right pleural effusion	7	Pericardial drain	Cured

2012	8/53/m	Cattle, pigs	Blood	Fever, cough, dyspnea	Renal tx	NA	NA	No	RUL dense consolidation	15	No	Cured

2003	9/25/f	Motor vehicle accident with soil debris	Tissue	Left foot wound	NA	NA	NA	No	NA	3 weeks	Amputation	Cured

2005	10/53/m	Unknown	Sputum	Fever, cough, dyspnea, weight loss	NA	NA	NA	No	RUL cavitary lesion	Unknown	No	Expired^‡^

2007	11/79/f	Unknown	Blood	Fever	NA	NA	NA	No	LUL mass lesion, bilateral infiltrates	Unknown	Central line removed	Expired^§^

2011	12/42/m	Unknown	Tissue	Right great toe wound	NA	NA	NA	No	NA	1	Amputation	Cured

^*∗*^HIV: human immune deficiency virus; tx: transplant; ART: antiretroviral therapy; RUL: right upper lobe; LUL: left upper lobe; LLL: left lower lobe; NA: not applicable.

^†^ *R. equi* bacteremia recurred after 9 months and the patient expired.

^‡^Lost to follow-up and expiredafter 15 months from unknown causes.

^§^Lost to follow-up and expired after 6 months from unknown causes.

**Table 2:** Organ systems involved in infection with *R. equi* in immunocompromised and immunocompetent patients in Europe and the United States.

Immune status	Europe^*∗*^		United States^†^	
	Pulmonary ± extrapulmonary	Extrapulmonary only	Pulmonary ± extrapulmonary	Extrapulmonary only
Immunocompromised	107/113^‡^ (95%)	6/113 (5%)	21/22 (95%)	1/22 (5%)
Immunocompetent	0	0	5/14 (36%)	9/14 (64%)^§^

^*∗*^From Arlotti et al. [11], Donisi et al. [12], Topino et al. [13], and Torres-Tortosa et al. [16].

^†^From Scott et al. [14], Verville et al. [15], and the 12 cases in Central Kentucky reported in the present study.

^‡^Number of patients with the organ involvement/total number of patients.

^§^ *P* = 0.0002 when compared to immunocompromised patients in the US and *P* < 0.0001 when compared to immunocompromised patients in Europe.

**Table 3:** Comparison of organ system involvement, sites of clinical isolation, and radiographic findings in *R. equi* infection in the seven studies under analysis.

Studies	Number of patients	Site of isolation^*∗*^			Organ system involved		Radiographic findings	
		Respiratory specimen (%)	Blood (%)	Other^†^	Pulmonary ± extrapulmonary (%)	Extrapulmonary only (%)	Pneumonia on imaging	Cavitary lesion (%)
Europe								
Donisi et al. [12]	12	4 (25)	10 (63)	2 (12)	9 (75)	3 (25)	9	5 (56)
Arlotti et al. [11]	24	21 (51)	13 (32)	7 (17)	24 (100)	0 (0)	24	18 (75)
Torres-Tortosa et al. [16]	67	64 (52)	34 (27)	26 (21)	65 (97)	2 (3)	65	45 (49)
Topino et al. [13]	10	5 (38)	8 (62)	0 (0)	9 (90)	1 (10)	9	6 (67)

Subtotal	113	94 (48)	65 (34)	35 (18)	107 (95)	6 (5)	107	74 (69)

United States								
Verville et al. [15]	12	8 (53)	5 (33)	2 (13)	8 (67)	4 (33)	8	6 (75)
Scott et al. [14]	12	6 (35)	7 (41)	4 (24)	8 (67)	4 (33)	8	7 (88)
University of Kentucky	12	7 (47)	5 (33)	3 (20)	10 (83)	2 (17)	10	4 (40)

Subtotal	36	21 (45)	17 (36)	9 (19)	26 (72)	10 (28)	26	17 (65)

Total	149	115 (48)	82 (34)	44 (18)	133 (89)	16 (11)	133	91 (68)

^*∗*^Total numbers of specimens are more than numbers of patients as *R. equi* was isolated from a variety of specimens.

^†^Bone, joint fluid, abscess, wound, pleura and pericardial fluid, liver, brain, cerebral spinal fluid, stool, and skin.

**Table 4:** Comparison of mortality in *R. equi*-infected HIV^+^ patients in pre-HAART and HAART era in the seven studies under analysis^*∗*^.

Studies	Total HIV patients	Diagnosed before 1997 (pre-HAART era)	Diagnosed in or after 1997 (HAART era)	Patients died (%) (pre-HAART era)	Patients died (%) (HAART era)
Europe^*∗*^	113	93	20	52 (56)	1 (5)

United States^†^	18	12	6	7 (58)	1 (17)

Total	131	105	26	59 (56)	2 (8)

^*∗*^From Arlotti et al. [11], Donisi et al. [12], Topino et al. [13], and Torres-Tortosa et al. [16].

^†^From the cases in the Central Kentucky reported in the present study, Scott et al. [14], and Verville et al. [15].

**Table 5:** Comparison of antibiotic susceptibilities of *R. equi* isolated from patients in Europe and the United States in the six studies under analysis^*∗*^.

Antibiotic	Europe				Subtotal (%)	United States		Subtotal (%)	*P* value	Total (%)
	Donisi et al. [12]	Arlotti et al. [11]	Torres-Tortosa et al. [16]	Topino et al. [13]		Scott et al. [14]	U of Kentucky			
*Clarithromycin*	NA	NA	NA	6/6^†^	6/6 (100)	NA	6/6	6/6 (100)	NS	12/12 (100)
*Vancomycin*	9/9	21/21	60/60	8/9	98/99 (99)	9/9	10/10	19/19 (100)	NS	117/118 (99)
*Rifampin*	1/2	21/21	47/48	6/8	75/79 (95)	NA	6/7	6/7 (86)	NS	81/86 (94)
*Amikacin*	NA	9/9	22/22	4/6	35/37 (95)	9/9	6/7	15/16 (94)	NS	50/53 (94)
*Erythromycin*	6/9	24/24	53/58	6/6	89/97 (92)	9/9	5/5	14/14 (100)	NS	103/111 (93)
*Teicoplanin*	8/8	9/9	NA	6/8	23/25 (92)	NA	NA	NA	NA	23/25 (92)
*Imipenem*	2/2	17/21	41/42	7/7	67/72 (93)	NA	4/6	4/6 (67)	NS	71/78 (91)
*Netilmicin*	8/8	8/9	NA	4/5	20/22 (91)	NA	NA	NA	NA	20/22 (91)
*Gentamicin*	NA	15/15	40/47	5/7	60/69 (87)	9/9	5/5	14/14 (100)	NS	74/83 (89)
*Ciprofloxacin*	6/9	11/19	47/50	8/9	72/87 (83)	9/9	6/6	15/15 (100)	NS	87/102 (85)
*Chloramphenicol*	NA	NA	12/18	6/6	18/24 (75)	9/9	NA	9/9 (100)	NS	27/33 (82)
*Ceftriaxone*	4/5	NA	NA	4/8	8/13 (62)	NA	3/6	3/6 (50)	NS	11/19 (58)
*Cotrimoxazole*	2/7	15/24	17/38	1/7	35/76 (46)	9/9	6/6	15/15 (100)	<0.0001^‡^	50/91 (55)
*Tetracycline*	0/8	6/18	15/22	2/5	23/53 (43)	9/9	0/1	9/10 (90)	0.0127^§^	32/63 (51)
*Amox-Clav*	NA	5/5	12/33	0/4	17/42 (40)	NA	3/6	3/6 (50)	NS	20/48 (42)
*Cefotaxime*	NA	NA	13/32	1/2	14/34 (41)	NA	NA	NA	NA	14/34 (41)
*Ampicillin*	NA	3/18	7/41	0/5	10/64 (16)	0/9	NA	0/9 (0)	NS	10/73 (14)
*Clindamycin*	1/10	NA	6/33	0/2	7/45 (16)	0/9	NA	0/9 (0)	NS	7/54 (13)
*Cephalothin*	0/4	2/20	NA	0/3	2/27 (7)	2/9	NA	2/9 (22)	NS	4/36 (11)
*Penicillin*	0/4	1/23	2/40	0/7	3/74 (4)	0/9	1/3	1/12 (8)	NS	4/86 (5)
*Oxacillin*	0/8	0/15	NA	0/7	0/30 (0)	0/9	NA	0/9 (0)	NS	0/39 (0)

^*∗*^NA: not applicable; NS: not significant.

^†^Number of clinical isolates susceptible to the antibiotic/total number of clinical isolates tested.

^‡^Comparison of the antibiotic susceptibility between Europe and the US, corrected *P* < 0.0018.

^§^Comparison of the antibiotic susceptibility between Europe and the US, corrected *P* = 0.229.
